# Study on the association of the microstructure and bone metabolism in the osteoporotic femoral head

**DOI:** 10.1007/s11033-023-08505-2

**Published:** 2023-07-21

**Authors:** Cheng Wang, Peng Wang, Feng Li, Yang Li, Minwei Zhao, Hui Feng, Haoye Meng, Junyang Li, Peng Shi, Jiang Peng, Hua Tian

**Affiliations:** 1 Department of Orthopaedics/Engineering Research Center of Bone and Joint Precision Medicine, Ministry of Education /Beijing Key Laboratory of Spinal Disease Research, Peking University Third Hospital, 49 North Garden Road, Haidian District, Beijing, 100191 China; 2 Institute of Orthopaedics, Beijing Key Laboratory of Regenerative Medicine in Orthopedics/Key Laboratory of Musculoskeletal Trauma & War Injuries PLA/The Fourth Medical Center of the General Hospital of People’s Liberation Army, Beijing, 100853 China; 3 Department of Electronic Engineering, Ocean University of China, Qingdao, China; 4 Centre for Robotics and Automation, Shenzhen Research Institute of City University of Hong Kong, Shenzhen, China

**Keywords:** Osteoporosis, Femoral head, Bone metabolism, Bone microstructure

## Abstract

**Background:**

We compared the bone microstructure and metabolism of the femoral heads in patients with osteoporosis (OP) and non-OP patients to investigate the pathologic mechanism of OP and guide clinical treatment.

**Methods and results:**

From January 2020 to June 2021, we obtained femoral head samples from 30 patients undergoing hip replacement due to femoral neck fracture. All patients were women aged approximately 67 to 80 years (mean age, 74 years). According to the dual-energy X-ray results, the femoral head samples were divided into the OP (T< − 2.5) and non-OP (T > − 1.5) groups. Microcomputed tomography scanning, bone metrology analysis, hematoxylin and eosin staining, and Masson’s trichrome staining were used to compare the local bone trabecular microstructure changes. Quantitative reverse transcription PCR was performed to identify changes in the osteogenesis-related genes and the osteoclast-related genes in specific regions to reflect osteogenic and osteoclastic activities. Femoral heads with OP showed significant changes in the local bone microstructure. Bone density, bone volume fraction, and the number and thickness of the bone trabeculae decreased. Local bone metabolism was imbalanced in the areas with microstructural changes in femoral heads with OP, with increased osteoclast activity and decreased osteoblast activity.

**Conclusions:**

Deterioration of bone microstructure is closely related to abnormal bone metabolism associated with the activity of osteoblasts and osteoclasts in osteoporotic femoral heads. Promoting bone formation by improving local bone metabolism, enhancing osteogenic activity and inhibiting osteoclast activity may be a promising way of preventing local OP and osteoporotic fractures.

## Introduction

Osteoporosis (OP) is a common orthopedic disease mainly affecting the elderly [1]. It is a systemic and metabolic disease of the skeletal system characterized by decreased bone mass and strength, destruction of the bone microstructure, and bone fragility [2]. Pathological fracture is the most severe consequence of OP, and it is often the first symptom and main reason for OP patients’ visits [3]. OP imposes a heavy burden on families and society by causing disability or even death in elderly patients. Therefore, there is an urgent need to reduce the incidence of OP, prevent the bone mass reduction and the decline in bone mechanical strength, and reduce the incidence of fractures [4]. However, OP is a complex disease involving many factors, and its pathogenesis has not been fully elucidated. Spinal compression and hip and distal radius fractures are the most common osteoporotic fractures [5–7]. Why these sites are particularly prone to fractures is unclear, and the changes in local bone microstructure are also unknown.

The mainstream view is that the imbalance in bone metabolism is the main major cause of OP. Under normal conditions, osteogenesis and osteoclasts regulate and influence each other, which exists in a state of dynamic balance [8, 9]. Osteoclasts secrete chemicals, cytokines, and enzymes to dissolve the bone matrix and continuously dissociate minerals [10, 11]. Osteoblasts, which secrete collagen and other matrix materials to provide a fibrous framework for mineral deposition, are mainly responsible for bone formation; mineralized osteoid become normal bone tissue [12, 13]. However, the mechanisms underlying bone metabolism imbalance and how this imbalance relates to the changes in the bone microstructure leading to OP and osteoporotic fractures remain unclear.

This study explored the correlation between bone microstructure changes and bone metabolic alterations in OP patients. We collected osteoporotic and non-osteoporotic femoral head samples from patients undergoing femoral neck fracture surgery. The femoral heads were subjected to comprehensive imaging and pathological and molecular biological analyses to learn more about the underlying mechanisms of OP. Investigating bone microstructure changes and local bone metabolism alterations helps to promote future prevention and treatment of OP and osteoporotic fractures.

## Materials and methods

### Sample collection

From January 2020 to June 2021, we collected femoral head specimens and clinical data from 30 patients undergoing hip replacement due to femoral neck fracture. All patients were female and approximately aged 67 to 80 (average age of 74 years). The inclusion criterion was patients with femoral neck fracture requiring joint replacement. The exclusion criteria were as follows: osteoarthritis (OA) of the hip joint; osteonecrosis of the femoral head; rheumatic immune diseases; tumor or other malignant diseases; complications including systemic severe internal diseases; and diabetes or related diseases affecting bone metabolism. The project was reviewed by the Peking University Third Hospital Medical Science Research Ethics Committee (IRB00006761-M2021216).

## Specimen grouping

According to the 2011 diagnostic criteria of the World Health Organization (WHO), a T value > − 1 was defined as normal, a T value from − 1 to − 2.5 was osteopenia, and a T value of < − 2.5 was OP, as determined by dual-energy X-ray absorptiometry (DXA). We defined a T value < − 2.5 as OP and a T value > − 1.5 as non-OP to ensure the accuracy of the results, and samples with a T value between − 2.5 and − 1.5 were excluded. The samples obtained were divided into the OP and non-OP groups based on these values. Each group included an identical number of samples of 15.

## Detection methods

### Microcomputed tomography (micro-CT) and bone metrology analysis

All thirty femoral head specimens were fully scanned by micro-CT (GE, Chicago, IL, USA), with a spatial resolution of 27 μm × 27 μm × 27 μm and a single scan time of 88 min. We used Microview software (GE, Chicago, IL, USA) for image processing, including three-dimensional reconstruction and osteometric analysis of the femoral head specimens. The region of interest (ROI) was located in the central position of the femoral head in the coronal position, and the selection size of osteometric analysis was 10 mm × 10 mm × 10 mm, while that of two-dimensional panoramic image reconstruction was 10 mm × 10 mm × 2.5 mm. Based on the selection of the ROI for osteometric analysis, the software automatically calculated the following bone mineral parameters: bone mineral density (BMD), bone volume fraction (bone volume/total volume; BV/TV), trabecular plate number (TB.n), trabecular plate thickness (TB.th), and trabecular spacing (TB.sp).

## Specimen preparation

**Fig. 1:**
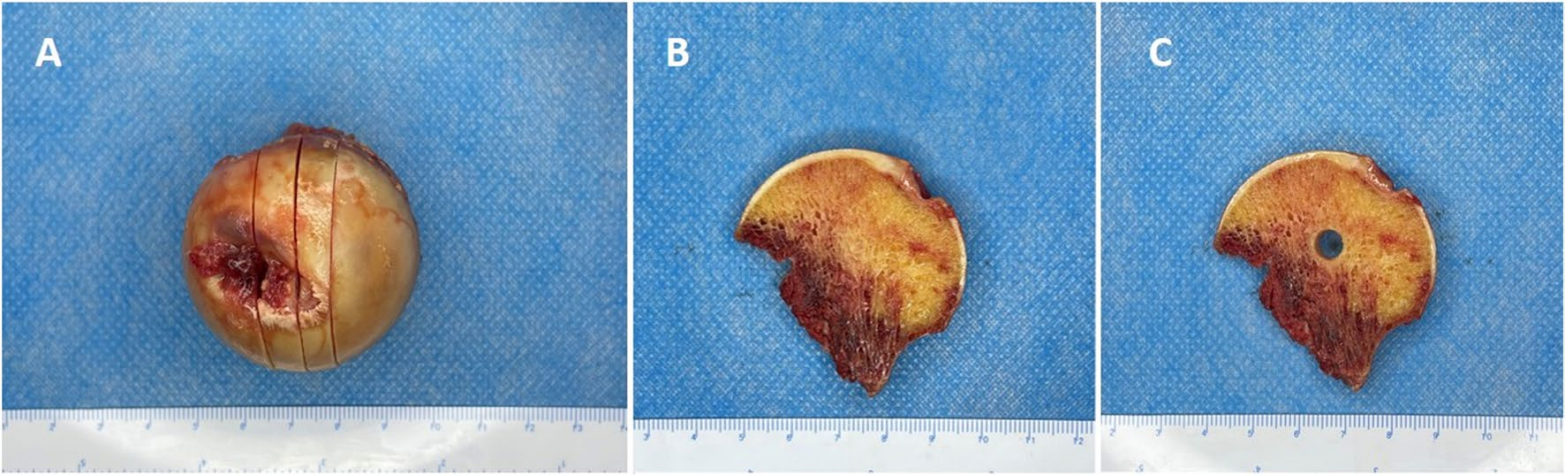
Femoral head specimen preparation. **A** The femoral heads were sawn into four parts along the coronal axis. **B**, **C** The special area in which the bone microstructure had changed was chosen to analyze bone metabolism

After micro-CT scanning, the femoral heads were sawn into four parts along the coronal axis (Fig. 1). The thickness of the middle two bone sections was 5 mm, and the middle two parts were subjected to pathological and molecular biological analysis. It should be noted that we chose a special area to analyze bone metabolism, in which the microstructure markedly changed according to the micro-CT results.

## Pathological staining

The sections of the femoral head in the two groups were fixed for one week and then decalcified for at least one month. After the sections became soft, they were dehydrated, embedded in paraffin, and cut into slices. The slices were placed in processing cassettes and dehydrated through a serial alcohol gradient. Before staining, the slices were dewaxed in xylene and washed in PBS. The sections were then stained with hematoxylin and eosin (HE) and Masson’s trichrome staining. Finally, slices were dehydrated through increasing concentrations of ethanol and xylene. Histological changes in the femoral head sections were observed after mounting and scanning.

## Quantitative reverse transcription PCR (qRT‒PCR)

We sampled bone tissue from the central region of the femoral head sections of the OP and non-OP groups, as the central region showed significant bone structure changes based on the micro-CT results. Bone tissue was powdered in liquid nitrogen before RNA extraction for qRT‒PCR. We selected the osteogenesis-related genes alkaline phosphatase (ALP), osteocalcin (BGLAP), and Osterix and the osteoclast-related genes nuclear factor of activated T cells 1 (NFATC1), acid phosphatase 5 (ACP5), tartrate-resistant cathepsin K (CTSK), and chloride voltage-gated channel 7 (CLCN7) to compare the osteoblast and osteoclast activities between the two groups (n = 6).

## Statistical analysis

Statistical analysis was performed using SPSS 22.0 statistical software (SPSS Inc., Chicago, IL, USA). The F test was used to compare the real-time PCR and bone measurement data. One-way ANOVA was used to compare the groups, with the least significant difference (LSD) test applied for post hoc analysis. P values < 0.05 were considered significant.

## Results

### Micro-CT and bone histomorphometry analyses

**Fig. 2:**
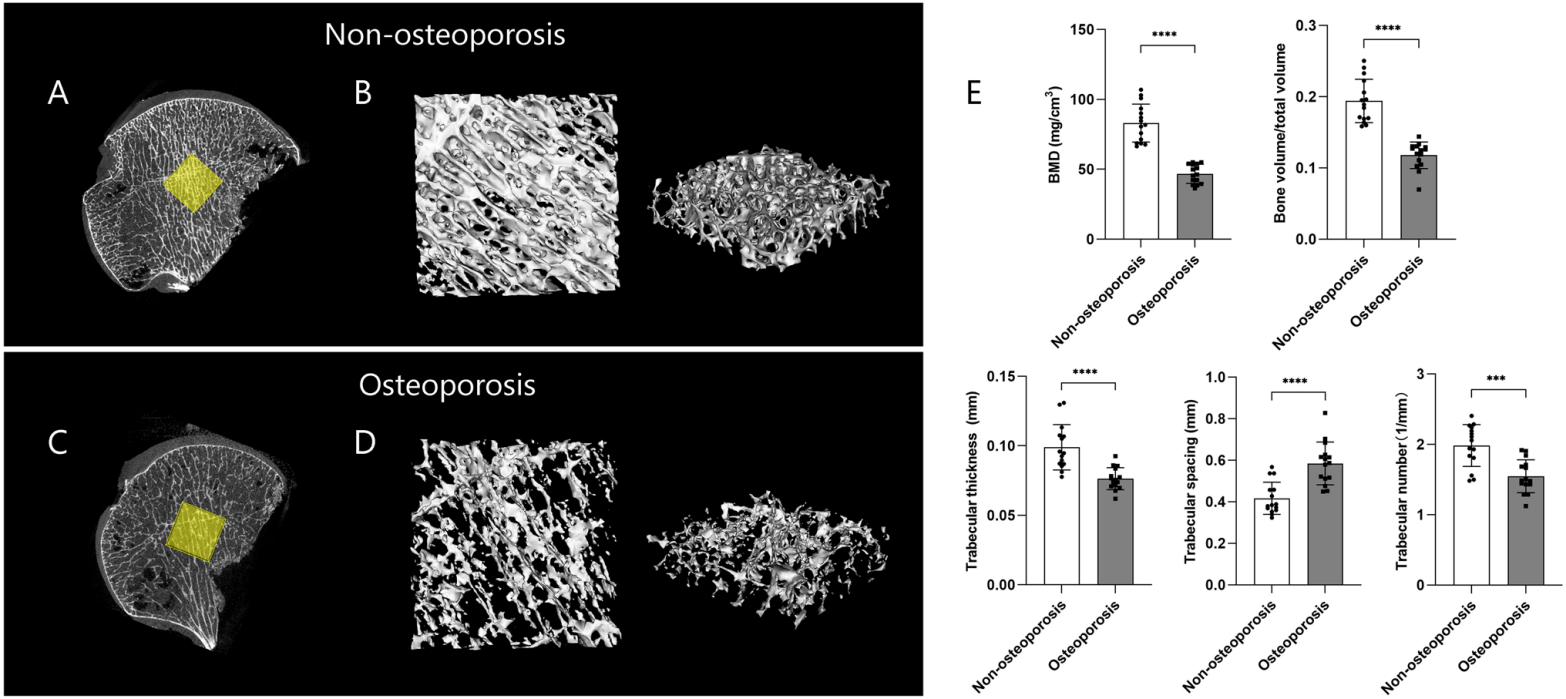
Results of micro-CT and bone histomorphometry analyses of femoral head samples from the osteoporos is and non-osteoporosis groups. For each group, n = 6. **A** Two-dimensional panoramic image of the femoral head of the nonosteoporosis group; the yellow area indicates the region of interest. **B** Three-dimensional reconstruction of the femoral head region of interest in the nonosteoporosis group. **C** Two-dimensional panoramic image of the femoral head in the osteoporosis group; the yellow area indicates the region of interest. **D** Three-dimensional reconstruction of the femoral head region of interest in the osteoporosis group. **E** Bone histomorphometry analysis results, including bone density, bone volume fraction, bone trabecular thickness, separation, and number. The error bars given with each data set is the standard devision. P value < 0.05 is considered significant. (***indicates P < 0.001. ****indicates P < 0.0001)

Micro-CT scans of the OP and non-OP femoral head specimens showed that both were spherical. However, the bone cortex of the OP femoral heads was thin, the trabecular gap was larger, the trabecular density was lower, and the trabecular bone was thinner and fewer in number compared with the non-OP femoral heads. The morphological parameters differed significantly between the two groups. Compared with the non-OP group, the BMD and BV/TV of bone tissue in the OP group were significantly reduced (P < 0.05). Furthermore, the TB.n and TB.th were significantly smaller in the OP group (P < 0.05), while the TB.sp was significantly larger (P < 0.05) (Fig. 2).

## Pathological results

**Fig. 3:**
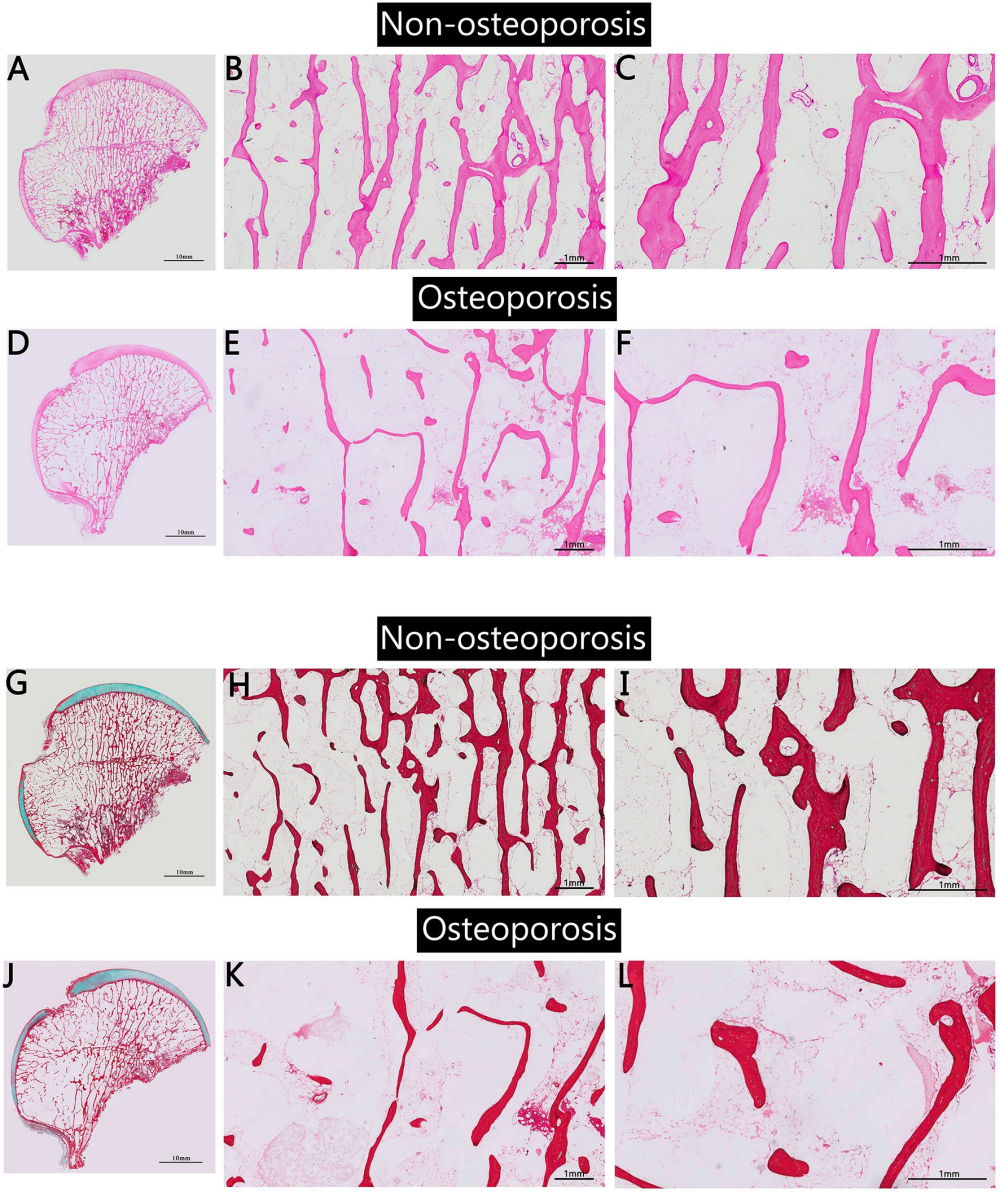
**A**–**F** HE staining and **G**–**L** Masson’s trichrome staining of nonosteoporotic and osteoporotic femoral head samples. The image shows the femoral head and field of view under ×100 and ×200 magnification

Compared with the non-OP group, the bone density of the cancellous bone in the OP group changed significantly. The bone trabeculae were thinner, and the continuity was disrupted; many free bone fragments and loss of normal arched bridge-like structure were observed. The intraosseous arterial and venous vascular network decreased significantly, representing low blood perfusion in the OP femoral head. The proportion of empty bone lacuna in trabecular bone was significantly increased, while the number of osteoblasts around the trabecular bone was decreased, suggesting that the regeneration of osteocytes and osteoblasts was inhibited (Fig. 3).

## qRT‒PCR results

**Fig. 4:**
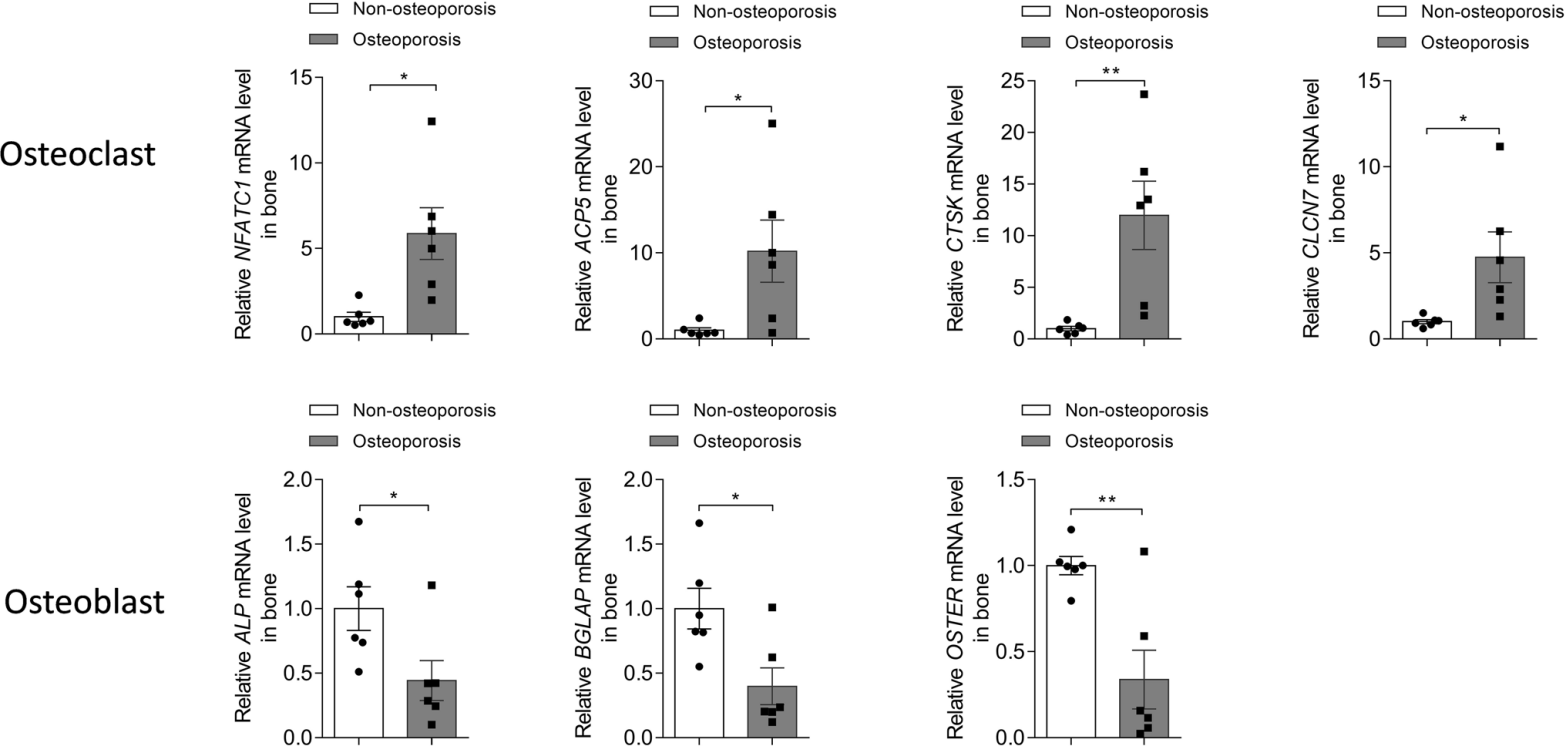
Quantitative real-time PCR analyses of osteoblast- and osteoclast-related genes in the OP and non-OP groups. Osteoblast-associated genes included ALP, BGLAP, and OSTER, and osteoclast-associated genes included NFATc1, ACP5, CTSK and CLCN7. For each group, n = 6. All data are means ± SDs. The control group used was the non-OP group, and ΔΔCt was calculated using the non-OP group as a control. P value < 0.05 is considered significant. (*indicates P < 0.05. **indicates P < 0.01)

According to the pathological staining and micro-CT scanning results, we selected the region where the local bone structure changed significantly and detected the gene expression related to bone metabolism in this region. qRT‒PCR revealed that the expression of NFATC1, ACP5, CTSK, and CLCN7 in the OP group was significantly increased (P < 0.05). However, the expression of ALP, BGLAP, and OSTER in the OP group was significantly decreased (P < 0.05) (Fig. 4).

## Discussion

The human skeleton provides protection and structural support for soft tissue, bone resorption and osteogenesis homeostasis to maintain normal skeletal metabolic activity [14]. Bone mass decreases with age, and the bone tissue microstructure undergoes changes. Cortical bone becomes thinner, and a reduction in the number and thickness of trabecular bones is evident. These substantial structural changes significantly affect local bone mechanical properties [15, 16]. The interior of the femoral head contains a large amount of cancellous bone, and fragility fractures rarely occur inside the femoral head. Therefore, few studies have examined the correlation between bone microstructure changes and bone metabolism in osteoporotic femoral heads.

Previous studies have performed comparative analyses of osteoporotic and OP femoral heads and analyzed the bone microstructure and mineral content. Tamimi et al. [17] compared 31 osteoporotic and 42 osteoarthritic femoral heads and found that the osteoarthritic femoral heads had higher BMD, BV/TV, TB.th, and TB.n values. Thus, they concluded that BMD was positively correlated with BV/TV and TB.n in the osteoarthritic femoral head. Conversely, this trend was not found in the osteoporotic femoral head. Therefore, the internal bone microstructure may not show the same changes in different osteoporotic femoral heads. Chu et al. [18] analyzed patients with both OP and OA disease, focusing on the impact of OP on the progression of OA and the correlation between the two conditions. The results showed that in the subchondral bone of patients with both OP and OA, abnormal bone remodeling resulted in microstructure changes and worse biomechanical properties compared with the OA group, which affected the transfer of load stress from the cartilage to subchondral bone and accelerated the progression of OA. The authors believed that changes in the subchondral bone affected the development of OA in patients with both OP and OA. Moreover, improving the quality of subchondral bone with bone metabolizers helped to attenuate the progression of OA. In other words, abnormal changes in bone metabolism were the key factor in OP progression.

Bone remodelling was the predominant metabolic process regulating bone structure and function during adult life, which was carried out by two key cell types: osteoclasts and osteoblasts [19, 20]. Osteoclasts are the principal resorptive cell of bone, while osteoblasts are specialised bone forming cells that synthesise bone matrix and finally differentiate into osteocytes or bone lining cells. Under physiological conditions, osteoblast-mediated bone formation and osteoclast-mediated bone resorption are maintained in a balance to ensure continuous renewal of bone tissue and to promote bone injury repair. The biological characteristic of OP is net loss of bone caused by bone resorption exceeding the formation of new bone. Bone microstructure changes caused by the bone loss are believed to be related to changes in local bone metabolism [21]. Laura et al. [22] found that an excessively high bone remodeling rate led to OP, which was related to estrogen deficiency, aging, oxidative stress, genetic factors, mechanical loading, and micro-damage. Estrogen deficiency affects circulating levels of cytokines such as IL-1, TNF-α, IL-6 and granulocyte macrophage colony stimulating factor. Xie et al. [23] found that the neuropeptide Y1 receptor played an essential regulatory role in bone metabolism and was a potential therapeutic target to prevent OP and osteoporotic fractures. Y1 receptor antagonists significantly improved the osteoporotic microstructure of ovariectomized (OVX) rats by promoting the expression of RUNX2 and OPG and inhibiting the expression of RANKL and MMP9 in the bone marrow. Notably, the interactions between osteoblasts and osteoclasts is an integral element in bone remodeling, but other cells also play an important part. Kular [24] et al. concluded that OP and other bone diseases occurred because of the failure of multicellular communication within the basic multicellular unit, which included the osteoclasts, the osteoblasts, the osteocytes, the bone lining cells and the capillary blood supply. Many immune cells were shown to directly or indirectly influence bone cells via factors including OPG/RANKL, inflammatory cytokines such as IL-6 and TNFα and other mediators secreted by immune cells [25].

This study analyzed femoral head samples from patients who suffered femoral neck fracture and then underwent joint replacement. The femoral heads were divided into the OP and non-OP groups based on the differences in clinical BMD examination results. The OP femoral heads were subjected to detailed and comprehensive tests, including micro-CT scans and histological and molecular biological analyses, to observe the changes in internal microstructure and bone metabolism in the bone tissue. We explored the relationship between bone microenvironment changes and bone structure changes and aimed to propose a possible method to reduce the incidence of osteoporotic fractures by regulating the local microenvironment.

We found that bone loss was obvious in osteoporotic femoral heads. The trabecular tissue of the OP group was characterized by thinned thickness, widened space, sparse arrangement and many free bone fragments. In addition, the expression of the NFATC1, ACP5, CTSK, and CLCN7 genes in the OP group was significantly increased compared with that in the non-OP group, while the expression of the ALP, BGLAP, and OSTER genes was significantly decreased. This result indicated that bone resorption was significantly greater than bone formation in osteoporotic femoral heads and that there was an inability of osteoblasts to maintain a sufficient rate of bone formation. Moreover, enhanced resorption of osteoclasts causes a greater resorption cavity, leading to the lack of a platform for osteoblasts to rebuild the bone. Therefore, bone metabolism imbalance is directly related to bone microstructure changes, which affect intraosseous biomechanics and ultimately lead to osteoporotic fractures after external trauma.

## Conclusions

For high-risk groups, we recommend that bone density screening be routinely performed in sites that are prone to osteoporotic fractures. Clinical BMD examination is helpful to understand the changes in bone microstructure in patients with OP. Deterioration of bone microstructure is closely related to local abnormal bone metabolism associated with the activity of osteoblasts and osteoclasts. Further studies will be needed to determine whether bone structure can be improved and OP and osteoporotic fractures prevented by regulating local bone metabolism with drugs.

## Data Availability

The data that support the findings of this study are available from the corresponding author, upon reasonable request.
